# Clinical performance of two commercial PCR assays for the detection of macrolide resistance in *Mycoplasma pneumoniae*

**DOI:** 10.1128/jcm.01491-25

**Published:** 2026-02-13

**Authors:** Nadège Hénin, Alicia Silvant, Marie Gardette, Carla Balcon, Jennifer Guiraud, Cécile Bébéar, Sabine Pereyre

**Affiliations:** 1 Univ. Bordeaux CNRS, UMR 5234 Fundamental Microbiology and Pathogenicity, Bordeaux, France; 2 Bacteriology Department, CHU Bordeaux, Bordeaux, France; Cleveland Clinic, Cleveland, Ohio, USA

**Keywords:** *Mycoplasma pneumoniae*, macrolides, antimicrobial resistance, 23S rRNA, PCR commercial kits

## Abstract

Macrolides are the first-line treatment for *Mycoplasma pneumoniae* infections; however, macrolide resistance has been reported. We evaluated the clinical performance of two commercial assays for detecting macrolide-resistant *M. pneumoniae*: the LightMix Modular *Mycoplasma Macrolide* (TIB Molbiol) and the *Mycoplasma Pneumoniae* and Macrolides-Resistant Strain Nucleic Acid Test Kit (Mole Bioscience), using 23S rRNA Sanger sequencing as the reference method. Eight *M. pneumoniae* strains, 10 non-*M*. *pneumoniae Mycoplasma* strains, and 237 clinical samples were tested. Overall, the Mole Bioscience kit failed to detect *M. pneumoniae* in 27.7% of positive samples, whereas the TIB Molbiol kit missed only 5.1%. The clinical sensitivities for detecting macrolide resistance–associated mutations in clinical samples were 90.9% for the TIB Molbiol kit and 81.5% for the Mole Bioscience kit. All false wild-type results corresponded to samples harboring a mutation at position 2067 (*M. pneumoniae* numbering) of the 23S rRNA gene. The clinical specificities were 97.9% and 94.4% for the TIB Molbiol and Mole Bioscience kits, respectively. Notably, the TIB Molbiol kit also detected 23S rRNA mutations in *Mycoplasma genitalium* and *Mycoplasma amphoriforme*, whereas the Mole Bioscience kit yielded false-resistant results in high-load specimens. In conclusion, these commercial kits offer a convenient approach for detecting macrolide resistance in routine clinical practice. The Mole Bioscience kit demonstrated limited sensitivity for *M. pneumoniae* detection, restricting its utility for resistance identification, and neither assay reliably detected resistance-associated mutations at position 2067. The global *M. pneumoniae* reemergence highlights the need for advanced assays detecting all macrolide resistance-associated mutations.

**IMPORTANCE:**

*Mycoplasma pneumoniae* is a leading cause of bacterial respiratory tract infections, and a marked resurgence was reported worldwide in autumn 2023. Macrolides are the recommended first-line treatment for *M. pneumoniae* infections; however, resistance rates vary widely across regions and may compromise therapeutic efficacy. Rapid identification of macrolide resistance is therefore critical to ensure appropriate patient management. Because culture-based methods are slow and have limited sensitivity, molecular assays have become central to both pathogen detection and resistance determination. However, few simple and reliable commercial assays have been developed and evaluated for the detection of macrolide resistance in routine diagnostic laboratories. In this study, we evaluated the clinical performance of two real-time PCR–based commercial assays for detecting macrolide resistance–associated mutations in *M. pneumoniae*. Our findings support the implementation of rapid molecular tools to guide timely therapeutic decisions, including the early use of second-line antimicrobials when resistance is detected.

## INTRODUCTION

*Mycoplasma pneumoniae* is a leading cause of bacterial respiratory tract infections, particularly community-acquired pneumonia, alongside *Streptococcus pneumoniae*; notably, it is most prevalent in school-aged children and young adults (1). Following a complete disappearance of *M. pneumoniae* infections during and immediately after the COVID-19 pandemic, a resurgence was documented in autumn 2023 across Europe, Asia, the Americas, and Oceania (2–4).

Macrolides remain the first-line therapy for *M. pneumoniae* infections (5). However, resistance rates exhibit considerable geographic variation (5, 6). The prevalence is highest in the Western Pacific region (53.4%), with particularly alarming rates in China (79.5%), while remaining comparatively low in Europe (5.1%) (6). Because conventional culture methods are limited by low sensitivity and prolonged turnaround times, molecular techniques are now widely used both for *M. pneumoniae* detection and identification of macrolide resistance–associated mutations.

Macrolide resistance in *M. pneumoniae* is primarily mediated by mutations in domain V of the 23S rRNA gene (5). The A2063G substitution (*M. pneumoniae* numbering) is the most common, conferring high-level resistance in clinical isolates, followed by A2064G (5, 6). Other substitutions, including A2063C, A2067G, A2067C, C2617A, and C2617G, occur less frequently, and mutations at position 2617 are only associated with low-level resistance to macrolides and related antibiotics (5). Mutations such as A2063G and A2064G may result in macrolide treatment failure (5, 7, 8). Therefore, assays that enable rapid detection of resistance–associated mutations are essential to guide timely therapeutic decisions and facilitate the early use of second-line antimicrobials in macrolide-resistant *M. pneumoniae* infections.

Only a limited number of commercial assays have been developed and evaluated for the detection of macrolide resistance–associated mutations in *M. pneumoniae* (9–12). To address this gap, we assessed the performance of two real-time PCR–based commercial assays for the detection of such mutations in clinical samples: the research-use-only (RUO) LightMix Modular *Mycoplasma Macrolide* Kit (TIB Molbiol, Berlin, Germany) and the Communauté Européenne *In Vitro* Diagnostic Medical Devices (CE-IVD)–marked *Mycoplasma Pneumoniae* and Macrolides-Resistant Strain Nucleic Acid Test (Mole Bioscience, Hangzhou, China). The clinical sensitivity and specificity of these assays for identifying 23S rRNA mutations associated with macrolide resistance were evaluated against 23S rRNA Sanger sequencing, used as the reference standard.

## MATERIALS AND METHODS

### Strains

Eight *M. pneumoniae* strains were used: two wild-type (WT) reference strains, M129 (ATCC 29342) and FH (ATCC 15531); five clinical isolates carrying 23S rRNA mutations (13) from the collection of the Bacteriology Department, Bordeaux University Hospital, France; and one *in vitro*–selected mutant, E3 (14) (Table 1). In addition, 10 strains of other *Mycoplasma* species were included: *Mycoplasma fermentans* PG18 (ATCC 19989), *Mycoplasma genitalium* G37 (ATCC 33530), *Mycoplasma hominis* PG21 (ATCC 23114), *Mycoplasma orale* CH 19299 (ATCC 23714), *Mycoplasma penetrans* GTU54 (ATCC 55252), *Mycoplasma salivarium* PG20 (ATCC 23064), *Ureaplasma parvum* S3 (ATCC 27815), *Ureaplasma urealyticum* S8 (ATCC 27618), 1 WT clinical isolate of *Mycoplasma amphoriforme* from the Bordeaux University Hospital collection, and 1 *M. genitalium in vitro*–selected mutant harboring the A2059G substitution (*Escherichia coli* numbering), mutant J10 (15).

**TABLE 1:** Mutations in the 23S rRNA gene among 137 *M. pneumoniae*-positive samples and the eight *M. pneumoniae* strains studied^*e*^

Mutations in the 23S rRNA gene^*a*^	*M. pneumoniae*-positive clinical specimens	*M. pneumoniae* strains
WT	100	2^*b*^
A2063C	0	1
A2063G	23	1
A2063G and A2063C	1	0
A2064G	3	1^*c*^
A2064G and A2067G and A2067C	1	0
A2067C	4	1
A2067C and A2067G	1	0
A2067G	1	0
C2617A	0	1^*d*^
C2617G	3	1
Total	137	8

^*a*^ *M. pneumoniae* numbering.

^*b*^ *M. pneumoniae* reference strains M129 (ATCC 29342) and FH (ATCC 15531).

^*c*^ Strain B3996 (13).

^*d*^ *In vitro*–selected *M. pneumoniae* mutant E3 (14).

^*e*^ WT, wild-type.

All strains were cultured by inoculating 200 µL of thawed stock into 1.8 mL of Hayflick medium supplemented with glucose and incubated until a color change of the medium was observed (16).

### Clinical samples

In total, 237 remnants of clinical specimens received at the Bacteriology Department, Bordeaux University Hospital, France, and stored at −80°C were retrospectively collected (Table 2). These comprised 100 consecutive *M. pneumoniae*-negative samples collected between December 2023 and January 2024, 100 consecutive *M. pneumoniae*–positive samples with the WT 23S rRNA gene collected between November 2023 and February 2024 (Table 1), and 37 *M. pneumoniae*-positive samples harboring 23S rRNA mutations, collected between 2008 and March 2024, representing all mutated specimens identified during this period in the Bordeaux Bacteriology Department (Table 1).

**TABLE 2:** Characteristics of the 237 clinical specimens analyzed

Specimens	*M. pneumoniae*–negative	*M. pneumoniae*–positive		Total
		No mutation in 23S rRNA	Mutation in 23S rRNA	
ENT^*a*^ swabs	71	26	6	103
Nasopharyngeal secretions	9	60	10	79
Respiratory tract samples^*b*^	13	12	11	36
Bronchoalveolar liquids	7	2	10	19
Total	100	100	37	237

^*a*^ ENT, ear, nose, and throat.

^*b*^ Respiratory tract specimens include sputum, tracheal secretions, and bronchial secretions.

The 237 clinical samples included ear, nose, and throat (ENT) swabs, nasopharyngeal secretions, respiratory tract samples, and bronchoalveolar lavage fluids (Table 2). Of these, 105 were obtained from women and 132 from men. The mean patient age was 34 years (10 days–96 years).

### DNA extraction

Nucleic acids were extracted from 200 µL of clinical specimens or strain cultures using the MagNA Pure 96 DNA and Viral NA Small Volume Kit on the MagNA Pure 96 Instrument (Roche Diagnostics, Meylan, France), yielding an elution volume of 100 µL. Prior to extraction, 10 µL of the LightMix Modular PhHV spiked Extraction Control (Roche Diagnostics/TIB Molbiol) was added according to the manufacturer’s instructions. For the Mole Bioscience kit, 0.25 µL of the internal control was added to the DNA extract prior to amplification after consulting the manufacturer. DNA extracts obtained from cultured strains were diluted 1:100 before downstream processing.

### Detection of *M. pneumoniae* and macrolide resistance-associated mutations

An in-house TaqMan real-time PCR assay targeting the *M. pneumoniae* P1 cytadhesin gene was performed as previously described, with a total of 50 amplification cycles (17). For four *M. pneumoniae*–negative specimens, amplification of the 16S rRNA gene followed by Sanger sequencing was performed to investigate other *Mycoplasma* species that had been suspected through melting curve analysis using the TIB Molbiol kit (18). For *M. pneumoniae* 23S rRNA sequencing, a 262 bp fragment was amplified from 5 µL of DNA, as previously reported (19). PCR products were submitted to Eurofins Genomics (Köln, Germany) for Sanger sequencing. The resulting sequences were analyzed with BioEdit software (version 7.2.5; Ionis Pharmaceuticals, Carlsbad, CA, USA) and compared with the WT *M. pneumoniae* M129 reference sequence (GenBank accession number NC_000912.1).

### Detection of macrolide resistance using commercial kits

Table 3 presents the main characteristics of the two evaluated assays.

**TABLE 3:** Main characteristics of the two commercial kits

Kits	LightMix Modular *Mycoplasma Macrolide* (TIB Molbiol)	*Mycoplasma Pneumoniae* and Macrolides-Resistant Strain Nucleic Acid Test Kit (Mole Bioscience)
PCR technology	FRET^*a*^	TaqMan
*M. pneumoniae* detection (targeted gene, if specified)	Not included	Included (P1 gene)
23S rRNA macrolide resistance-associated mutations detected^*b*^	Not specified	A2063(2058)G, A2064(2059)G
Validated specimen types	Vaginal swabs; urine, throat, or nasopharyngeal swabs; nasal or endotracheal aspirates; sputum or lavage	Sputum
Validated DNA extraction methods	MagNA Pure (Roche)	Nucleic acid extraction kits using the 32M, 96M, or FAST96 instruments (Mole Bioscience) or boiling method
Input sample volume	5 or 10 µL of DNA extract	5 µL of DNA extract
Internal control	Not included	Included: synthetic oligonucleotide
Positive controls	Included: 23S rRNA fragment with no mutation	Included: strong and weak positive control (P1 gene and 23S rRNA harboring the A2063G mutation), quality control (23S rRNA with no mutation)
Number of tests/kits	96	16
Hands-on time (min)	15–45, depending on sample number	15–45, depending on sample number
Test turnaround time^*c*^	1 h 20 min	1 h 30 min
Validated amplification instruments	LightCycler 480 Instrument; LightCycler 480 II Instrument; Cobas z 480 Analyzer open channel (Roche Diagnostics)	Stratagene MX 3000P (Agilent Technologies, Santa Clara, CA, USA); ABI 7500 (Applied Biosystems, Waltham, MA, USA), CFX96 (Bio-Rad)
Data analysis software	LightCycler 480 Software release 1.5.1.62	Bio-Rad CFX Maestro 1.1 (4.1.2433.1219)
List price/reaction (€), excluding taxes	4.10 € (including the LightCycler Multiplex DNA Master reagent [ref 07 339 585 001] not provided in the kit)	13.17 €
CE marking^*d*^	RUO	CE-IVD

^*a*^ FRET, fluorescence resonance energy transfer.

^*b*^ *M. pneumoniae* numbering, with *E. coli* numbering in parentheses.

^*c*^ DNA extraction not included.

^*d*^ RUO, research use only; CE-IVD, Communauté Européenne *In Vitro* Diagnostic Medical Devices.

#### LightMix Modular *Mycoplasma Macrolide* (TIB Molbiol)

The TIB Molbiol kit is a RUO PCR assay, based on melting curve analysis, designed to detect mutations in the 23S rRNA gene of *M. pneumoniae* and *M. genitalium*. The 23S rRNA macrolide resistance–associated mutations targeted are not disclosed in the manufacturer’s instructions. Assays were performed with 5 µL of DNA extract on the LightCycler 480 Instrument (Roche Diagnostics). Data were analyzed using LightCycler 480 Software (version 1.5.1.62; Roche Diagnostics). According to the manufacturer’s instructions, the melting temperature (*T*_*m*_) of the probe hybridized to the WT sequence ranges between 66°C and 69°C, whereas the *T*_*m*_ for probes hybridized to mutated sequence was <64°C. No information was provided for results within the 64°C–66°C interval.

#### *Mycoplasma Pneumoniae* and Macrolides-Resistant Strain Nucleic Acid Test Kit (Mole Bioscience)

Mole Bioscience kit is a CE-IVD–marked TaqMan PCR assay designed to detect *M. pneumoniae* by targeting the P1 cytadhesin gene and two resistance-associated mutations in the 23S rRNA gene (A2063G and A2064G). The kit includes extraction and amplification controls. Assays were performed with 5 µL of DNA extract on a CFX96 Real-Time PCR System (Bio-Rad Laboratories, Hercules, CA, USA). Data were analyzed using CFX Maestro 1.1 (version 4.1.2433.1219; Bio-Rad). According to the manufacturer’s instructions, *M. pneumoniae* detection was considered positive for cycle threshold (*C*_*T*_) values ≤ 35.01, and macrolide resistance was considered present for samples with *C*_*T*_ values ≤ 35.33.

### Data analysis

Clinical sensitivity and specificity for detecting macrolide resistance–associated mutations were calculated, with corresponding 95% confidence intervals (CIs). Overall agreement was assessed using the kappa (*κ*) statistic.

## RESULTS

### Evaluation of the two commercial kits on clinical isolates and reference strains

Eight *M. pneumoniae* strains and 10 strains from other *Mycoplasma* species were evaluated with both assays. As expected, the TIB Molbiol kit, designed to detect macrolide resistance–associated mutations in both *M. pneumoniae* and *M. genitalium* species, produced amplification with melting curve profiles for all corresponding strains (Table 4). No amplification was observed for the other *Mycoplasma* species tested, with the exception of the *M. amphoriforme* clinical isolate. Using the Mole Bioscience kit, all eight *M. pneumoniae* strains were correctly identified, and no amplification occurred with the 10 non-*M*. *pneumoniae* strains.

**TABLE 4:** Performance of the two commercial kits on reference strains and clinical isolates^*e*^^*,^f^*^

	TIB Molbiol		Mole Bioscience	
Strains (23S rRNA sequence)	Amplification	Resistance detection (23S rRNA 2063–2067 region)	*M. pneumoniae* detection	Resistance detection (23S rRNA 2063–2067 region)
Mpn FH (WT)	Positive	WT	Positive	**R^*d*^**
Mpn M129 (WT)	Positive	WT	Positive	**R^*d*^**
Mpn 7477 (A2063G^*a*^)	Positive	R	Positive	R
Mpn 3996 (A2064G^*a*^)	Positive	R	Positive	R
Mpn 4357 (A2063C^*a*^)	Positive	R	Positive	**WT**
Mpn 7182 (A2067C^*a*^)	Positive	**WT**	Positive	R^*d*^
Mpn 4318 (C2617G^*a*^)	Positive	WT	Positive	**R^*d*^**
Mpn E3 (C2617A^*a*^)	Positive	WT	Positive	**R^*d*^**
Mg G37 (WT)	Positive^*c*^	WT^*c*^	Negative	–^*g*^
Mg J10 (A2072G^*b*^)	Positive^*c*^	R^*c*^	Negative	**R**
Mh PG21 (WT)	Negative	–	Negative	–
Up S3 (WT)	Negative	–	Negative	–
Uu S8 (WT)	Negative	–	Negative	–
Mf PG18 (WT)	Negative	–	Negative	–
Mo CH 19299(WT)	Negative	–	Negative	–
Mpe GTU54 (WT)	Negative	–	Negative	–
Ms PG20 (WT)	Negative	–	Negative	–
Ma 4527 (WT)	**Positive**	**R**	Negative	–

^*a*^ *M. pneumoniae* numbering.

^*b*^ *M. genitalium* numbering.

^*c*^ The TIB Molbiol kit is also intended to detect mutations in the 23S rRNA gene of *M. genitalium*.

^*d*^ Classified as resistant with 1:100 DNA dilution, but WT with 1:10,000 DNA dilution.

^*e*^ Discrepant results are in bold.

^*f*^ Mpn, *M. pneumoniae*; Mh, *M. hominis*; Up, *U. parvum*; Uu, *U. urealyticum*; Mg, *M. genitalium*; Mf, *M. fermentans*; Mo, *M. orale*; Mpe, *M. penetrans*; Ms, *M. salivarium*; Ma, *M. amphoriforme*; WT, wild-type; R, resistant; -, No resistance detection.

^*g*^ "–", Not applicable.

Using the TIB Molbiol kit, the WT control exhibited a lower *T*_*m*_ than expected (mean *T*_*m*_, 64.68°C [64.52°C–64.88°C]), that is, lower than the manufacturer’s specified range of 66°C–69°C. Similarly, all four *M. pneumoniae* strains without mutations in the 23S rRNA 2063–2067 region displayed *T*_*m*_ values between 64.53°C and 64.85°C, also outside the manufacturer’s specified range. Consequently, for subsequent analyses, specimens with a *T*_*m*_ consistent with the WT control and above 64°C were classified as macrolide-susceptible. Among the four *M. pneumoniae* strains harboring a mutation in the 23S rRNA 2063–2067 region, three yielded *T*_*m*_ values < 64°C and were correctly classified as macrolide-resistant, whereas the strain with the A2067C mutation was classified as susceptible (Table 4). Results for the WT and mutated *M. genitalium* strains were concordant as the TIB Molbiol kit is also designed to detect macrolide resistance-associated mutations in *M. genitalium*. By contrast, the *M. amphoriforme* clinical isolate was falsely classified as macrolide-resistant, despite lacking mutations in the 23S rRNA gene.

Using the Mole Bioscience kit, strains carrying the A2063G and A2064G mutations were correctly classified as macrolide-resistant. The strain harboring the A2063C mutation was misclassified as macrolide-susceptible (Table 4), reflecting a limitation of the assay, which is not designed to detect this mutation. Unexpectedly, the four strains without mutations in the 23S rRNA 2063–2067 region were falsely identified as macrolide-resistant. However, a significant discrepancy was observed between the *C*_*T*_ value for *M. pneumoniae* detection and that for mutation detection; the mean *C*_*T*_ difference was 9.9 in the falsely resistant WT strains compared to only 1.3 in the correctly identified mutated strains. This pattern was also evident for the A2067C strain and suggested non-specific amplification at high DNA concentration. To address this, the DNA extract dilution was increased from 1/100 to 1/10,000. At this higher dilution, the four WT strains were correctly classified as susceptible, and the A2067C strain shifted from resistant to WT classification. In addition, the *M. genitalium* J10 strain, which carries an A2059G substitution (corresponding to A2064G using *M. pneumoniae* numbering), yielded a resistance result; however, it was correctly classified as *M. pneumoniae*–negative by the kit.

### Performance for the detection of *M. pneumoniae* in clinical samples

In total, 237 clinical specimens were evaluated against the in-house PCR assay for *M. pneumoniae* detection. No invalid results were observed with the TIB Molbiol or the Mole Bioscience kit.

The TIB Molbiol kit is not designed to detect *M. pneumoniae* but solely to identify macrolide resistance–associated mutations. However, melting curve generation was observed in 4 ENT swabs of the 100 *M. pneumoniae*–negative specimens, suggesting limited analytical specificity. These included two WT-like and two mutation-like results. Subsequent 16S rRNA amplification and sequencing revealed *M. amphoriforme* in one WT-like sample and *Mycoplasma faucium* in one mutation-like sample, whereas no *Mycoplasma* species were identified in the remaining two. Among the 137 *M. pneumoniae*–positive samples, 7 (5.1%)—comprising 4 nasopharyngeal secretions and 3 ENT swabs—failed to yield a melting curve with the TIB Molbiol kit (Table 5). All of these samples had a low bacterial load, with in-house PCR *C*_*T*_ values ranging from 36.05 to 38.32.

**TABLE 5:** Performance of the two commercial kits for the detection of macrolide resistance compared to the 23S rRNA Sanger sequencing^*a*^

		23S rRNA sequencing result			Overall % agreement (95% CI), *κ* value	Sensitivity (%) (95% CI)	Specificity (%) (95% CI)
Commercial assays (manufacturer)	23S rRNA mutation detection results	Mutated within the 2063–2067 fragment	Not mutated within the 2063–2067 fragment	Total			
LightMix Modular *Mycoplasma Macrolide* (TIB Molbiol)	Detected	30	2	32	96.2 (91.3–98.4) *κ* = 0.90	90.9 (76.4–96.9)	97.9 (92.8–99.4)
	Not detected	3	95	98			
	NA	1	6	7			
	Total	34	103	137			
*Mycoplasma Pneumoniae* and Macrolides-Resistant Strain Nucleic Acid Test Kit (Mole Bioscience)	Detected	22	4	26	90.9 (83.6–95.1) *κ* = 0.77	81.5 (63.3–91.8)	94.4 (86.6–97.8)
	Not detected	5	68	73			
	NA	7	31	38			
	Total	34	103	137			

^*a*^ NA, not amplified; WT, wild type; CI, confidence interval.

Using the Mole Bioscience kit, the number of samples incorrectly classified as *M. pneumoniae*–negative was significantly higher than with the TIB Molbiol kit, totaling 38 (27.7%) specimens (*P* < 0.05, *χ*² test) (Table 5). These included 22 nasopharyngeal secretions, 10 ENT swabs, 4 respiratory tract specimens, and 2 bronchoalveolar lavages. All of these samples had a low bacterial load, with a mean *C*_*T*_ value of 36.47 (34.10–40.00) in the in-house PCR, compared with a mean *C*_*T*_ value of 30.84 (18.31–36.50) for correctly detected *M. pneumoniae*–positive samples. Among the 38 undetected samples, only 1 failed to amplify; the remaining were interpreted as negative because their *C*_*T*_ values exceeded 35.01. All 100 *M. pneumoniae*–negative specimens were correctly reported as negative. Finally, the Mole Bioscience kit demonstrated an overall percent agreement of 84.0% (95% CI, 78.8–88.1) with the in-house PCR assay for *M. pneumoniae* detection, corresponding to a *κ* value of 0.69. The positive percent agreement was 72.3% (95% CI, 64.2–79.1), whereas the negative percent agreement was 100% (95% CI, 96.3–100).

### Performance for the detection of macrolide resistance–associated mutations in clinical samples

In total, 137 *M. pneumoniae*-positive samples were concurrently tested using the 2 commercial assays and compared with 23S rRNA Sanger sequencing as the reference standard. The overall agreement with sequencing was 96.2% and 90.9% for the TIB Molbiol and the Mole Bioscience kits, respectively, with no statistically significant difference between the two (Table 5). The corresponding *κ* values were 0.90 and 0.77, respectively.

The clinical sensitivity for detecting 23S rRNA macrolide resistance–associated mutations was 90.9% for the TIB Molbiol kit and 81.5% for the Mole Bioscience kit (Table 5), with no statistically significant differences between the assays. Among the three samples incorrectly classified as macrolide-susceptible by the TIB Molbiol kit, one harbored the A2067C mutation, one the A2067G mutation, and one contained a mixture of A2067C and A2067G mutations. Similarly, the five samples falsely categorized as susceptible by the Mole Bioscience kit all harbored mutations at position 2067, comprising three with the A2067C mutation, one with the A2067G mutation, and one with a mixed population of both variants (Table 1).

The clinical specificity of the TIB Molbiol kit and the Mole Bioscience kit was 97.9% and 94.4%, respectively, with no significant difference between the two assays (Table 5). For the TIB Molbiol kit, two samples—one nasopharyngeal secretion and one ENT swab—were falsely identified as macrolide-resistant. In both cases, the observed *T*_*m*_ values (63.81°C and 63.7°C, respectively) were close to those of the 94 WT amplified samples (mean *T*_*m*_, 64.86°C [64.07°C–65.60°C]), whereas the mean *T*_*m*_ of the 30 concordant macrolide-resistant samples was significantly lower (58.65°C [57.88°C–62.23°C]). For the Mole Bioscience kit, four samples were falsely classified as macrolide-resistant. In these cases, a significant discrepancy was observed between the *C*_*T*_ values for *M. pneumoniae* detection (17.24–21.37) and those for mutation detection (30.83–35.28). This observation prompted retesting after a 1:100 dilution, which yielded the expected macrolide-susceptible results.

Finally, a supplemental analysis was performed with stratification by specimen type, including the 70 *M. pneumoniae*–positive nasopharyngeal secretions (Table S1), the 32 *M. pneumoniae*–positive ENT swabs (Table S2), the 23 *M. pneumoniae*–positive respiratory tract samples (Table S3), and the 12 *M. pneumoniae*–positive bronchoalveolar lavage fluids (Table S4). While only minor differences were observed overall for the TIB Molbiol kit, the Mole Bioscience kit showed notably poorer performance for respiratory tract samples, including sputum, tracheal, and bronchial secretions, with an overall percentage agreement and a kappa value of 73.7% and 0.48, respectively (Table S3), despite the validation of sputum use in the manufacturer’s instructions.

## DISCUSSION

We evaluated the performance of two commercial kits for detecting macrolide resistance–associated mutations in *M. pneumoniae*, a respiratory pathogen that has recently reemerged worldwide (2, 3). Several in-house methods have previously been reported, including PCR-restriction fragment length polymorphism (20), pyrosequencing (21, 22), real-time PCR with melting curve analysis (19), and real-time PCR with high-resolution melt analysis (23), with reported clinical sensitivities in the range of 72.6%–80.2%. To directly identify macrolide-resistant mutants in clinical samples, single-nucleotide polymorphism PCR assays (24) and nested PCR combined with single-strand conformation polymorphism and capillary electrophoresis (25) have also been developed. Despite these advances, the availability of simple, reliable commercial assays remains limited, although such tools are essential for routine diagnostic laboratories and for guiding clinical management of *M. pneumoniae* infections. The ResistancePlus MP assay (SpeeDx, Inc., Austin, TX, USA) demonstrated a sensitivity of 81.8% and a specificity of 99.45% (10); however, it was no longer available during the recent reemergence of *M. pneumoniae*. In Japan, two other commercial kits were evaluated: the Smart Gene assay (Mizuho Medy Co., Ltd., Tosu City, Saga, Japan), which achieved a sensitivity of 97.8% and specificity of 93.3% (9), and the GENECUBE Mycoplasma assay (TOYOBO Co., Ltd., Osaka, Japan), which demonstrated sensitivity and specificity of 100% (11). An earlier version of the TIB Molbiol kit, the “Lightmix *Mycoplasma* macrolide assay,” reported a sensitivity of 100% (95% CI 78.2–100) and a specificity of 100% (95% CI 97.5–100) for detecting macrolide resistance in respiratory samples, consistent with our findings (12).

The two kits evaluated here serve distinct purposes and differ in assay design, which may contribute to sensitivity variations. The Mole Bioscience kit is designed as a first-line assay for the simultaneous detection of *M. pneumoniae* and macrolide resistance–associated mutations, whereas the TIB Molbiol kit is intended as a second-line assay dedicated solely to the detection of macrolide resistance. Notably, only the Mole Bioscience kit carries CE-IVD marking, whereas the TIB Molbiol kit is designated for RUO; neither has Food and Drug Administration (FDA) clearance. Moreover, the TIB Molbiol kit does not disclose the specific mutation detected to the end user. Finally, in our study, the *T*_*m*_ values obtained from WT samples using the TIB Molbiol kit did not fully align with the manufacturer’s recommended range. Consequently, the WT control provided with the kit proved essential for *T*_*m*_ comparison and accurate interpretation of macrolide-susceptible results.

Using the TIB Molbiol kit, 5.1% of positive samples failed to amplify, corresponding to low-load specimens. In contrast, with the Mole Bioscience kit, a marked lack of sensitivity for *M. pneumoniae* detection was observed and subsequently impeded the detection of macrolide resistance–associated mutations. In particular, results were unavailable for 27.7% of samples (38 specimens; Table 5). Although these 38 undetected specimens contained a low *M. pneumoniae* load, amplification was in fact achieved for 37 of them; however, their *C*_*T*_ values exceeded the manufacturer’s cutoff of 35.01 and were classified as *M. pneumoniae*–negative. This raises concern regarding the appropriateness of the *C*_*T*_ threshold, as its strict application significantly reduced the positive agreement for *M. pneumoniae* detection. An increase in the *C*_*T*_ threshold should therefore be considered. Notably, in this study, a cutoff of 38.01 would have enabled the accurate detection of 35 out of the 38 undetected samples. Moreover, among these 38 false-negative samples, one was reported positive for macrolide resistance. This occurred because the *C*_*T*_ value for *M. pneumoniae* detection (35.1) was above the cutoff of 35.01, whereas the *C*_*T*_ value for resistance detection (35.0) was below the cutoff of 35.33. The manufacturer’s instructions do not clarify how such discrepancies should be interpreted. An alternative explanation for this finding could be the presence of other mutated *Mycoplasma* species in the specimen, as the *M. genitalium* J10 mutant strain was detected as macrolide-resistant in the absence of *M. pneumoniae* detection (Table 4).

Using the Mole Bioscience kit, discrepancies in mutation detection were observed in high-load strains and clinical samples. This issue affected four strains and four samples (Tables 4 and 5), which were falsely classified as macrolide-resistant. Accurate results were obtained after dilution, suggesting a saturation phenomenon or cross-reactivity at high DNA concentration. This methodological limitation raises significant concerns for routine diagnostics, as it may lead to erroneous identification of macrolide resistance in high-load samples, resulting in the unnecessary use of more toxic antibiotics in patient management.

The TIB Molbiol kit is intended solely for second-line testing of samples already confirmed as *M. pneumoniae* or *M. genitalium* positive. However, this design presents limitations for the detection of macrolide resistance in *M. pneumoniae*, as *M. genitalium* may rarely colonize the oropharyngeal cavity. Notably, some studies have reported throat colonization rates ranging from 0.5% to 1% (26–28). Considering the high global prevalence of macrolide resistance in *M. genitalium* (33.3% between 2018 and 2021 [29]), this kit may overestimate macrolide-resistant *M. pneumoniae*. Another factor undermining its specificity is its ability to amplify *M. amphoriforme* DNA, as demonstrated in our findings (Table 4). In addition, 4 of the 100 *M. pneumoniae*-negative clinical samples produced amplification signals with melting curves; 2 of these contained either *M. amphoriforme* or *M. faucium*. The detection of these species is not unexpected, as *M. faucium* is a commensal of the human oropharynx, and *M. amphoriforme* has been described as a potential respiratory pathogen (30–32). In cases of co-infection with *M. pneumoniae*, the accuracy of the TIB Molbiol assay is compromised, as the detected macrolide resistance–associated mutations may originate from non-*M*. *pneumoniae* mycoplasma species.

Neither of the two kits accurately detected macrolide resistance–associated mutations at position 2067 as all three and five samples falsely detected as macrolide-susceptible using the TIB Molbiol and the Mole Bioscience kits harbored a mutation at this position, respectively. This lack of detection is related to the assay design of the kits, which likely does not cover the 2067 mutation position. Indeed, excluding the six clinical samples with mutations at position 2067 yielded higher sensitivities for both kits: 100% (95% CI 87.9–100) and 100% (95% CI 84.5–100), with *κ* values of 0.96 and 0.89, for the TIB Molbiol and Mole Bioscience kits, respectively, whereas the specificities remained unchanged (data not shown). The A2067G substitution has been associated with increased josamycin and pristinamycin MICs in *M. pneumoniae* and in *M. genitalium*, the closest phylogenetic relative of *M. pneumoniae* (14, 15, 33). To our knowledge, the A2067C substitution has never been reported in *M. pneumoniae* in the literature. In our study, the clinical isolate harboring this mutation, obtained in 2016 from an immunocompromised patient with persistent *M. pneumoniae* infection, exhibited significantly elevated minimum inhibitory concentrations of josamycin (8 µg/mL) and pristinamycin (4 µg/mL); however, no increase in the minimum inhibitory concentrations of erythromycin or azithromycin was observed compared to the susceptible M129 reference strain (unpublished personal data). In the present study, single mutations at position 2067 were present in 16.2% of the 37 samples analyzed, a proportion that is clinically relevant. These findings emphasize the need to incorporate detection of such mutations in future kit development.

In conclusion, the Mole Bioscience kit demonstrated limited sensitivity for *M. pneumoniae* detection, thereby compromising its ability to accurately identify macrolide resistance. The TIB Molbiol kit lacks specificity for *M. pneumoniae*, as it can amplify DNA from other *Mycoplasma* species, increasing the risk of falsely attributing macrolide resistance–associated mutations to *M. pneumoniae*. Moreover, neither assay reliably detected resistance-associated mutations at position 2067. Considering the global reemergence of *M. pneumoniae* and the high prevalence of macrolide resistance in certain regions, the development of novel, highly specific assays capable of detecting all macrolide resistance–associated mutations is needed.

## SUPPLEMENTAL MATERIAL

Tables S1 to S4 jcm.01491-25-s0001.docx Performance of the two commercial kits according to specimen types.

ASM does not own the copyrights to Supplemental Material that may be linked to, or accessed through, an article. The authors have granted ASM a non-exclusive, world-wide license to publish the Supplemental Material files. Please contact the corresponding author directly for reuse.

## References

[1] Yun KW, Wallihan R, Desai A, Alter S, Ambroggio L, Cohen DM, El-Assal O, Marzec S, Florin TA, Keaton M, Shah SS, Ruddy RM, Sharpe S, Leber AL, Everhart K, Mejias A, Ramilo O, Children’s Hospitals Initiative for Research in Pneumonia. 2022. Clinical characteristics and etiology of community-acquired pneumonia in US children, 2015-2018. Pediatr Infect Dis J 41:381–387. doi:10.1097/INF.0000000000003475 35143427

[2] Meyer Sauteur PM, Beeton ML, European Society of Clinical Microbiology and Infectious Diseases (ESCMID) Study Group for Mycoplasma and Chlamydia Infections (ESGMAC), and the ESGMAC Mycoplasma pneumoniae Surveillance (MAPS) study group. 2024. Pneumonia outbreaks due to re-emergence of Mycoplasma pneumoniae. Lancet Microbe 5:e514. doi:10.1016/S2666-5247(23)00406-8 38342111

[3] ESGMAC MAPS Study Group. 2025. Global spatiotemporal dynamics of Mycoplasma pneumoniae re-emergence after COVID-19 pandemic restrictions: an epidemiological and transmission modelling study. Lancet Microbe 6:101019. doi:10.1016/j.lanmic.2024.101019 40024259

[4] Meyer Sauteur PM, Beeton ML, ESGMAC the ESGMAC MAPS Study Group. 2023. Mycoplasma pneumoniae: gone forever? Lancet Microbe 4:e763. doi:10.1016/S2666-5247(23)00182-9 37393927

[5] Pereyre S, Goret J, Bébéar C. 2016. Mycoplasma pneumoniae: current knowledge on macrolide resistance and treatment. Front Microbiol 7:974. doi:10.3389/fmicb.2016.00974 27446015 PMC4916212

[6] Kim K, Jung S, Kim M, Park S, Yang HJ, Lee E. 2022. Global trends in the proportion of macrolide-resistant Mycoplasma pneumoniae infections: a systematic review and meta-analysis. JAMA Netw Open 5:e2220949. doi:10.1001/jamanetworkopen.2022.20949 35816304 PMC9274321

[7] Cheong K-N, Chiu SS, Chan BW-K, To KK-W, Chan EL-Y, Ho P-L. 2016. Severe macrolide-resistant Mycoplasma pneumoniae pneumonia associated with macrolide failure. J Microbiol Immunol Infect 49:127–130. doi:10.1016/j.jmii.2014.11.003 25556047

[8] Ding G, Zhang X, Vinturache A, van Rossum AMC, Yin Y, Zhang Y. 2024. Challenges in the treatment of pediatric Mycoplasma pneumoniae pneumonia. Eur J Pediatr 183:3001–3011. doi:10.1007/s00431-024-05519-1 38634891

[9] Kakiuchi T, Miyata I, Kimura R, Shimomura G, Shimomura K, Yamaguchi S, Yokoyama T, Ouchi K, Matsuo M. 2021. Clinical evaluation of a novel point-of-care assay to detect Mycoplasma pneumoniae and associated macrolide-resistant mutations. J Clin Microbiol 59:e0324520. doi:10.1128/JCM.03245-20 33910960 PMC8218770

[10] Leal SM, Totten AH, Xiao L, Crabb DM, Ratliff A, Duffy LB, Fowler KB, Mixon E, Winchell JM, Diaz MH, Benitez AJ, Wolff BJ, Qin X, Tang YW, Gonzalez M, Selvarangan R, Hong T, Brooks E, Dallas S, Atkinson TP, Zheng X, Dien Bard J, Waites KB. 2020. Evaluation of commercial molecular diagnostic methods for detection and determination of macrolide resistance in Mycoplasma pneumoniae. J Clin Microbiol 58:e00242-20. doi:10.1128/JCM.00242-20 32269102 PMC7269381

[11] Morinaga Y, Suzuki H, Notake S, Mizusaka T, Uemura K, Otomo S, Oi Y, Ushiki A, Kawabata N, Kameyama K, Morishita E, Uekura Y, Sugiyama A, Kawashima Y, Yanagihara K. 2020. Evaluation of GENECUBE Mycoplasma for the detection of macrolide-resistant Mycoplasma pneumoniae. J Med Microbiol 69:1346–1350. doi:10.1099/jmm.0.001264 33141009

[12] Wagner K, Imkamp F, Pires VP, Keller PM. 2019. Evaluation of Lightmix Mycoplasma macrolide assay for detection of macrolide-resistant Mycoplasma pneumoniae in pneumonia patients. Clin Microbiol Infect 25:383. doi:10.1016/j.cmi.2018.10.006 30391582

[13] Dégrange S, Cazanave C, Charron A, Renaudin H, Bébéar C, Bébéar CM. 2009. Development of multiple-locus variable-number tandem-repeat analysis for molecular typing of Mycoplasma pneumoniae. J Clin Microbiol 47:914–923. doi:10.1128/JCM.01935-08 19204097 PMC2668363

[14] Pereyre S, Guyot C, Renaudin H, Charron A, Bébéar C, Bébéar CM. 2004. In vitro selection and characterization of resistance to macrolides and related antibiotics in Mycoplasma pneumoniae. Antimicrob Agents Chemother 48:460–465. doi:10.1128/AAC.48.2.460-465.2004 14742195 PMC321523

[15] Le Roy C, Byambaa O, Guiraud J, Balcon C, Gillet L, Skov Jensen J, Bébéar C, Pereyre S. Oral poster presentation at STI and HIV 2025 World Congress. In Vitro Selection and Characterization of Resistance to Josamycin and Pristinamycin in Mycoplasma Genitalium. Montreal, Canada 10.1128/aac.00094-26 PMC13148045 41914618

[16] Waites KB, Bébéar CM, Roberston JA, Talkington DF, Kenny GE. 2001. Cumitech 34, Laboratory diagnosis of mycoplasmal infections. American Society for Microbiology, Washington, DC.

[17] Touati A, Benard A, Hassen AB, Bébéar CM, Pereyre S. 2009. Evaluation of five commercial real-time PCR assays for detection of Mycoplasma pneumoniae in respiratory tract specimens. J Clin Microbiol 47:2269–2271. doi:10.1128/JCM.00326-09 19403761 PMC2708483

[18] van Kuppeveld FJ, van der Logt JT, Angulo AF, van Zoest MJ, Quint WG, Niesters HG, Galama JM, Melchers WJ. 1992. Genus- and species-specific identification of mycoplasmas by 16S rRNA amplification. Appl Environ Microbiol 58:2606–2615. doi:10.1128/aem.58.8.2606-2615.1992 1381174 PMC195828

[19] Peuchant O, Ménard A, Renaudin H, Morozumi M, Ubukata K, Bébéar CM, Pereyre S. 2009. Increased macrolide resistance of Mycoplasma pneumoniae in France directly detected in clinical specimens by real-time PCR and melting curve analysis. J Antimicrob Chemother 64:52–58. doi:10.1093/jac/dkp160 19429926

[20] Matsuoka M, Narita M, Okazaki N, Ohya H, Yamazaki T, Ouchi K, Suzuki I, Andoh T, Kenri T, Sasaki Y, Horino A, Shintani M, Arakawa Y, Sasaki T. 2004. Characterization and molecular analysis of macrolide-resistant Mycoplasma pneumoniae clinical isolates obtained in Japan. Antimicrob Agents Chemother 48:4624–4630. doi:10.1128/AAC.48.12.4624-4630.2004 15561835 PMC529214

[21] Spuesens EBM, Hoogenboezem T, Sluijter M, Hartwig NG, van Rossum AMC, Vink C. 2010. Macrolide resistance determination and molecular typing of Mycoplasma pneumoniae by pyrosequencing. J Microbiol Methods 82:214–222. doi:10.1016/j.mimet.2010.06.004 20547188

[22] Spuesens EBM, Meijer A, Bierschenk D, Hoogenboezem T, Donker GA, Hartwig NG, Koopmans MPG, Vink C, van Rossum AMC. 2012. Macrolide resistance determination and molecular typing of Mycoplasma pneumoniae in respiratory specimens collected between 1997 and 2008 in The Netherlands. J Clin Microbiol 50:1999–2004. doi:10.1128/JCM.00400-12 22495561 PMC3372106

[23] Wolff BJ, Thacker WL, Schwartz SB, Winchell JM. 2008. Detection of macrolide resistance in Mycoplasma pneumoniae by real-time PCR and high-resolution melt analysis. Antimicrob Agents Chemother 52:3542–3549. doi:10.1128/AAC.00582-08 18644962 PMC2565909

[24] Ji M, Lee NS, Oh JM, Jo JY, Choi EH, Yoo SJ, Kim HB, Hwang SH, Choi SH, Lee SO, Kim MN, Sung H. 2014. Single-nucleotide polymorphism PCR for the detection of Mycoplasma pneumoniae and determination of macrolide resistance in respiratory samples. J Microbiol Methods 102:32–36. doi:10.1016/j.mimet.2014.04.009 24780151

[25] Lin C, Li S, Sun H, Zhao H, Feng Y, Cao L, Yuan Y, Zhang T. 2010. Nested PCR-linked capillary electrophoresis and single-strand conformation polymorphisms for detection of macrolide-resistant Mycoplasma pneumoniae in Beijing, China. J Clin Microbiol 48:4567–4572. doi:10.1128/JCM.00400-10 20861333 PMC3008430

[26] Berçot B, Charreau I, Rousseau C, Delaugerre C, Chidiac C, Pialoux G, Capitant C, Bourgeois-Nicolaos N, Raffi F, Pereyre S, Le Roy C, Senneville E, Meyer L, Bébéar C, Molina J-M, ANRS IPERGAY Study Group. 2021. High prevalence and high rate of antibiotic resistance of Mycoplasma genitalium infections in men who have sex with men: a substudy of the ANRS IPERGAY pre-exposure prophylaxis trial. Clin Infect Dis 73:e2127–e2133. doi:10.1093/cid/ciaa1832 33305785

[27] Calas A, Zemali N, Camuset G, Jaubert J, Manaquin R, Saint-Pastou C, Koumar Y, Poubeau P, Gerardin P, Bertolotti A. 2021. Prevalence of urogenital, anal, and pharyngeal infections with Chlamydia trachomatis, Neisseria gonorrhoeae, and Mycoplasma genitalium: a cross-sectional study in Reunion island. BMC Infect Dis 21:95. doi:10.1186/s12879-021-05801-9 33478403 PMC7818901

[28] Latimer RL, Shilling HS, Vodstrcil LA, Machalek DA, Fairley CK, Chow EPF, Read TR, Bradshaw CS. 2020. Prevalence of Mycoplasma genitalium by anatomical site in men who have sex with men: a systematic review and meta-analysis. Sex Transm Infect 96:563–570. doi:10.1136/sextrans-2019-054310 32341023

[29] Chua TP, Vodstrcil LA, Murray GL, Plummer EL, Jensen JS, Unemo M, Chow EPF, Low N, Whiley DM, Sweeney EL, Hocking JS, Danielewski JA, Garland SM, Fairley CK, Zhang L, Bradshaw CS, Machalek DA. 2025. Evolving patterns of macrolide and fluoroquinolone resistance in Mycoplasma genitalium: an updated systematic review and meta-analysis. Lancet Microbe 6:101047. doi:10.1016/j.lanmic.2024.101047 40147462

[30] Balish MF, Chopra-Dewasthaly R, Pereyre S, Ramirez AS, Viver T, Spergser J. 2024. Mycoplasma. *In* Gasparich GF (ed), Bergey's manual of systematics of archaea and bacteria. John Wiley & Sons.

[31] Day J, Afshar B, Rowlands RS, Umer TS, Windsor H, Paukner S, Jensen JS, Spiller OB, Chalker VJ, Beeton ML, ESCMID Study Group for Mycoplasma and Chlamydia Infections (ESGMAC). 2022. Phenotypic and genotypic antimicrobial susceptibility patterns of the emerging human respiratory pathogen Mycoplasma amphoriforme isolated from the UK and Denmark. J Antimicrob Chemother 77:3126–3129. doi:10.1093/jac/dkac293 36048620 PMC9616542

[32] Pereyre S, Renaudin H, Touati A, Charron A, Peuchant O, Hassen AB, Bébéar C, Bébéar CM. 2010. Detection and susceptibility testing of Mycoplasma amphoriforme isolates from patients with respiratory tract infections. Clin Microbiol Infect 16:1007–1009. doi:10.1111/j.1469-0691.2009.02993.x 19624516

[33] Guschin A, Ryzhikh P, Rumyantseva T, Gomberg M, Unemo M. 2015. Treatment efficacy, treatment failures and selection of macrolide resistance in patients with high load of Mycoplasma genitalium during treatment of male urethritis with josamycin. BMC Infect Dis 15:40. doi:10.1186/s12879-015-0781-7 25645440 PMC4318211

