# Supplementary material for: Clinical performance of two commercial PCR assays for the detection of macrolide resistance in Mycoplasma pneumoniae
Source: J Clin Microbiol. 2026 Feb 13;64(3):e01491-25. doi: 10.1128/jcm.01491-25 (PMC12977576; doi:10.1128/jcm.01491-25)
Supplement: Tables S1 to S4 — Performance of the two commercial kits according to specimen types. [file jcm.01491-25-s0001.docx]

**Table S1.** Performance of the two commercial kits for the detection of macrolide resistance compared to the 23S rRNA Sanger sequencing in the 70 *M. pneumoniae*-positive nasopharyngeal secretions.

|  |  | **23S rRNA sequencing result** | | | **Overall % agreement**  **(95% CI), κ value** | **Sensitivity (%)**  **(95% CI)** | **Specificity (%)**  **(95% CI)** |
| --- | --- | --- | --- | --- | --- | --- | --- |
| **Commercial assays (manufacturer)** | **23S rRNA mutation**  **detection results** | **Mutated within the 2063–2067 fragment** | **Not mutated within the 2063–2067 fragment** | **Total** |  |  |  |
| LightMix^®^ Modular *Mycoplasma Macrolide* (TIB Molbiol) | Detected | 8 | 1 | 9 | 97.0 (89.6-99.2)  κ = 0.87 | 88.9 (56.5-98.0) | 98.2 (90.7-99.7) |
|  | Not detected | 1 | 56 | 57 |  |  |  |
|  | NA | 0 | 4 | 4 |  |  |  |
|  | Total | 9 | 61 | 70 |  |  |  |
| *Mycoplasma pneumoniae* and Macrolides-resistant Strain Nucleic Acid Test Kit  (Mole Bioscience) | Detected | 4 | 1 | 5 | 93.8 (83.2-97.9)  κ = 0.69 | 66.7 (30.0-90.3) | 97.6 (87.7-99.6) |
|  | Not detected | 2 | 41 | 43 |  |  |  |
|  | NA | 3 | 19 | 22 |  |  |  |
|  | Total | 9 | 61 | 70 |  |  |  |

NA, not amplified; WT, wild type; CI, confidence interval.

**Table S2.** Performance of the two commercial kits for the detection of macrolide resistance compared to the 23S rRNA Sanger sequencing in the 32 *M. pneumoniae*-positive ENT swabs.

|  |  | **23S rRNA sequencing result** | | | **Overall % agreement**  **(95% CI), κ value** | **Sensitivity (%)**  **(95% CI)** | **Specificity (%)**  **(95% CI)** |
| --- | --- | --- | --- | --- | --- | --- | --- |
| **Commercial assays (manufacturer)** | **23S rRNA mutation**  **detection results** | **Mutated within the 2063–2067 fragment** | **Not mutated within the 2063–2067 fragment** | **Total** |  |  |  |
| LightMix^®^ Modular *Mycoplasma Macrolide* (TIB Molbiol) | Detected | 5 | 1 | 6 | 96.6 (82.8-99.4)  κ = 0.89 | 100 (56.6-100) | 95.8 (79.8-99.3) |
|  | Not detected | 0 | 23 | 23 |  |  |  |
|  | NA | 1 | 2 | 3 |  |  |  |
|  | Total | 6 | 26 | 32 |  |  |  |
| *Mycoplasma pneumoniae* and Macrolides-resistant Strain Nucleic Acid Test Kit  (Mole Bioscience) | Detected | 4 | 0 | 4 | 95.5 (78.2-99.2)  κ = 0.86 | 80.0 (37.6-96.4) | 100 (81.6-100) |
|  | Not detected | 1 | 17 | 18 |  |  |  |
|  | NA | 1 | 9 | 10 |  |  |  |
|  | Total | 6 | 26 | 32 |  |  |  |

NA, not amplified; WT, wild type; CI, confidence interval.

**Table S3.** Performance of the two commercial kits for the detection of macrolide resistance compared to the 23S rRNA Sanger sequencing in the 23 *M. pneumoniae*-positive respiratory tract samples^a^.

|  |  | **23S rRNA sequencing result** | | | **Overall % agreement**  **(95% CI), κ value** | **Sensitivity (%)**  **(95% CI)** | **Specificity (%)**  **(95% CI)** |
| --- | --- | --- | --- | --- | --- | --- | --- |
| **Commercial assays (manufacturer)** | **23S rRNA mutation**  **detection results** | **Mutated within the 2063–2067 fragment** | **Not mutated within the 2063–2067 fragment** | **Total** |  |  |  |
| LightMix^®^ Modular *Mycoplasma Macrolide* (TIB Molbiol) | Detected | 8 | 0 | 8 | 91.3 (73.2-97.6)  κ = 0.82 | 80.0 (49.0-94.3) | 100 (77.2-100) |
|  | Not detected | 2 | 13 | 15 |  |  |  |
|  | NA | 0 | 0 | 0 |  |  |  |
|  | Total | 10 | 13 | 23 |  |  |  |
| *Mycoplasma pneumoniae* and Macrolides-resistant Strain Nucleic Acid Test Kit  (Mole Bioscience) | Detected | 7 | 3 | 10 | 73.7 (51.2-88.2)  κ = 0.48 | 77.8 (45.3-93.7) | 70.0 (39.7-89.2) |
|  | Not detected | 2 | 7 | 9 |  |  |  |
|  | NA | 1 | 3 | 4 |  |  |  |
|  | Total | 10 | 13 | 23 |  |  |  |

^a^Respiratory tract samples include sputum, tracheal secretions, and bronchial secretions.

NA, not amplified; WT, wild type; CI, confidence interval.

**Table S4.** Performance of the two commercial kits for the detection of macrolide resistance compared to the 23S rRNA Sanger sequencing in the 12 *M. pneumoniae*-positive bronchoalveolar liquids.

|  |  | **23S rRNA sequencing result** | | | **Overall % agreement**  **(95% CI), κ value** | **Sensitivity (%)**  **(95% CI)** | **Specificity (%)**  **(95% CI)** |
| --- | --- | --- | --- | --- | --- | --- | --- |
| **Commercial assays (manufacturer)** | **23S rRNA mutation**  **detection results** | **Mutated within the 2063–2067 fragment** | **Not mutated within the 2063–2067 fragment** | **Total** |  |  |  |
| LightMix^®^ Modular *Mycoplasma Macrolide* (TIB Molbiol) | Detected | 9 | 0 | 9 | 100 (75.8-100)  κ = 1 | 100 (70.1-100) | 100 (43.9-100) |
|  | Not detected | 0 | 3 | 3 |  |  |  |
|  | NA | 0 | 0 | 0 |  |  |  |
|  | Total | 9 | 3 | 12 |  |  |  |
| *Mycoplasma pneumoniae* and Macrolides-resistant Strain Nucleic Acid Test Kit  (Mole Bioscience) | Detected | 8 | 0 | 8 | 91.7 (64.6-98.5)  κ = 0.8 | 88.9 (56.5-98.0) | 100 (43.9-100) |
|  | Not detected | 1 | 3 | 4 |  |  |  |
|  | NA | 0 | 0 | 0 |  |  |  |
|  | Total | 9 | 3 | 12 |  |  |  |

NA, not amplified; WT, wild type; CI, confidence interval.
